# Exploring the impact of reimbursement ratios on willingness to vaccinate: A mixed-effects modeling approach using panel data

**DOI:** 10.1080/21645515.2025.2609339

**Published:** 2026-03-03

**Authors:** Linbo Xie, Jie Xing, Mengsha Yan, Peiyao Li, Junfang Xu, Xin Fang, Ziting Guo, Min Yuan, Jiming Zhu

**Affiliations:** a Vanke School of Public Health, Tsinghua University, Beijing, China; b Institute for Healthy China, Tsinghua University, Beijing, China; c Department of Health Data Science, Anhui Medical University, Hefei, Anhui, China; d MOE Key Laboratory of Population Health Across Life Cycle, Hefei, Anhui, China; e Zhejiang University School of Medicine, Hangzhou, China; f T. H. Chan School of Public Health, Harvard University, Boston, MA, USA

**Keywords:** Childhood immunization, mixed-effect model, self-paid vaccines, machine learning, willingness to vaccinate, vaccine hesitancy

## Abstract

Vaccination remains one of the most cost-effective methods for disease prevention. However, utilization of self-paid vaccines, including EV71, varicella, influenza, and DTaP-IPV-Hib in this study, remains insufficient among children under six in China. To investigate the determinants of willingness to vaccinate (WTV) for self-paid vaccines and assess cost-WTV heterogeneity, we conducted structured-questionnaire surveys with 2212 randomly selected households in Hangzhou, each with at least one child under six. Multiple regression analysis was used to identify the key determinants of WTV, and a mixed-effect model was employed to analyze the correlation between vaccine cost and WTV, further segmenting the data with unsupervised clustering techniques. Our findings highlighted impact of vaccination cost as a pivotal factor influencing the WTV for self-paid vaccines. We categorized the population into four groups based on their sensitivity to vaccine cost. Families with one child, children aged 1–3 y, highly-educated parents, and higher socioeconomic status consistently exhibited high WTV. Our analysis offers targeted strategies to enhance vaccine uptake and improve immunization coverage.

## Introduction

Vaccination stands as one of the most cost-effective and vital public health interventions against infectious diseases, annually saving countless lives and preserving the well-being of humanity.^1–4^ The China National Immunization Program (NIP) is structured as a national-level public health program that aims to prevent and control infectious diseases through vaccination. The program is managed by the National Health Commission of China and is implemented at the provincial, city, and county levels. The China NIP provides free vaccines for a range of diseases, including polio, measles, mumps, rubella, pertussis, tetanus, diphtheria, hepatitis B, and tuberculosis.^5^ These vaccines are funded by the government and are provided to children at no cost. While the NIP provides many vaccines at no cost, there is a separate category of vaccines that require partial self-payment.^2,6^ These include vaccines for diseases such as influenza and pneumococcal disease, as well as combination vaccines like DTaP-IPV-Hib, which is a non-NIP vaccine that offers protection against diphtheria, tetanus, pertussis, polio, and Haemophilus influenzae type b (Hib). The cost of these vaccines varies depending on the type and brand, but generally ranges from a few hundred to over a thousand yuan per dose.^2^ In China, the coverage rates of self-paid vaccines for conditions such as DTaP-IPV-Hib in Hainan province (24.4% for at least one dose),^7^ hand, foot, and mouth disease (EV71 vaccine) in Zhejiang province (with a significant increase from 0.35% in 2015 to 30.45% in 2018),^8^ varicella in Chongqing,^9^ and influenza^10^ remained low considering China’s large population. In contrast, the coverage rates of NIP vaccines, such as BCG, hepatitis B, and measles-containing vaccines, have been consistently high, exceeding 95% since 2016.^2^ Despite these improvements, the coverage rates of non-NIP vaccines remain significantly lower than those of NIP vaccines, highlighting the need for further efforts to improve access and acceptance of self-paid vaccines.

The existing literature has predominantly focused on psychosocial factors associated with vaccination, indicating that knowledge, attitudes, and beliefs toward vaccines affect vaccination behaviors.^11–15^ Nevertheless, the substantial cost of vaccines could pose a significant obstacle to self-paid vaccination in China.^16–19^ A recent study surveying three provinces in eastern, central, and western China, identified economic factors as primary barriers to vaccination, particularly for the 7-valent pneumococcal conjugate and influenza vaccines.^20^ The survey conducted in Hainan Province also pointed out that socioeconomic disparities, cultural beliefs, worries regarding vaccine safety, and cost may contribute to the low coverage rate of the DTaP-IPV/Hib vaccine.^7^ The elevated expenses associated with self-financed vaccines likely hinder individuals’ decision to be vaccinated. Recognizing the financial barrier of vaccination, the Chinese government is actively contemplating subsidizing a portion of vaccination costs to increase the rates of vaccination.^21^ One key aspect is that including vaccines in insurance coverage or providing subsidies can significantly reduce the financial burden on patients. Consequently, it becomes crucial to assess how medical insurance reimbursement rates influence the WTV. Currently, there is a lack of research on the relationship between reimbursement ratios and economic efficiency. Understanding this connection could provide valuable insights to inform and improve decision-making processes regarding vaccination policy.

In this study, we analyze panel data concerning willingness-to-vaccinate scores for four predominantly self-paid vaccines (EV71, varicella, influenza and DTaP-IPV-Hib), gathered in varying proportions. By further implementing a mixed-effects model that correlates reimbursement rates with payment scores, we effectively segmented the population and uncovered distinct patterns of payment willingness across various groups. Our models provide data-driven insights that can inform local health authorities in developing more tailored vaccine reimbursement and communication strategies, while also serving as a methodological reference for similar studies in other regions.

## Materials and methods

### Study design, population and sampling

This study was conducted in Hangzhou, Zhejiang Province, which was selected as the pilot site based on data accessibility, system reliability, and operational feasibility. Zhejiang established a province-wide Immunization Information System (ZJIIS) as early as 2004, integrating all vaccination clinics across the province. The system has been validated for its accuracy in recording immunization coverage and thus provided a robust infrastructure for reliable data collection in this pilot investigation. To reflect economic diversity within the city, three districts/counties (Gongshu, Linping and Tonglu) were selected to represent high-, medium-, and low-economic levels, respectively. This stratified design enabled the study to capture intra-city socioeconomic variation and to evaluate the feasibility of using a digital immunization registry for assessing self-paid vaccine uptake.

The sample size was determined using the formula n = (Z_1_-α/2/δ)^2^  × p × (1 - p), with an error α = 0.05, Z_1_-α/2 = 1.96, and desired precision of δ = ± 2%. Based on a conservative estimate of 25% for the vaccination rate in Hangzhou and a non-response rate of 10.0%, the required number of surveyed caregivers of children was 2001. In practice, a larger sample size of 2212 was collected to improve the reliability of results.

**Figure 1.**
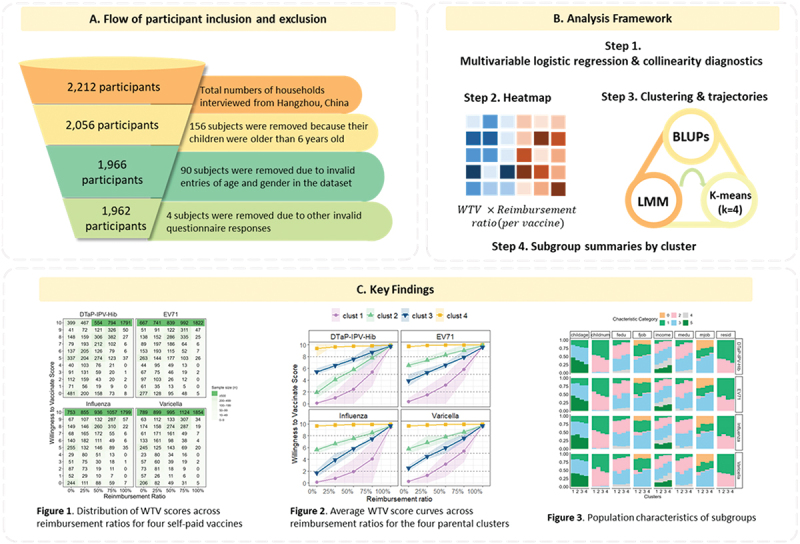
Flowchart of the current study, detailing sample inclusion and exclusion procedures (A), the stepwise analytical framework used in the analysis (B), and a visual summary of key findings derived from the modeling approaches (C).

To select 2212 households through the clinics without overlaps in households with multiple eligible kids, the following steps were taken: three districts/counties (i.e., Gongshu District, Linping District, and Tonglu County) were selected to represent high-, medium-, and low-economic levels respectively from a total of 13 districts (district/county/city) in Hangzhou. Subsequently, 1 to 2 vaccination points (community health centers, maternity childcare hospitals) from each sample district were randomly selected to conduct the survey. The medical staff in vaccination clinics who received standard training by our researchers conducted the data collection to ensure the quality of the survey. We invited all caregivers who took their children aged under 6 y to get vaccinated to participate in the survey. If a household had multiple eligible kids, only one child was randomly selected to participate in the survey. The study participants are parents and caregivers of children aged less than 6 y. Detailed flowchart from the study sample inclusion and exclusion, to the analytical method and main results is shown in Figure 1.

### Data cleaning and exclusion

After the initial recruitment, 2212 households were included. However, 156 participants (7.05%) reported that their child was over 6 y old and were excluded. Then, 238 surveys were excluded due to missing important sociodemographic or outcome variables. After that, 1 record with missing doctor recommendations was removed. Further, records with outliers in gender (not normal), father’s age ( >100), child’s age (≥200), number of children ( >6), medical history ( >5), and mother’s age ( >50) were excluded, leaving 1966 records. Finally, after excluding records with invalid responses that did not match the predefined options for the variables of pandemic timing (survey conducted during COVID-19 control measures), opening-hours coverage (extent to which clinics offer evening/weekend hours), and residence status (local vs. non-local hukou), 1962 valid records were retained.

Data acquisition utilized a paper-based structured questionnaire comprising four sections: intentions and reasons for self-paid vaccination, vaccine hesitancy, demographics of caregivers and children, and out-of-pocket WTV. The study also examined the proportion of self-payment for vaccinations. Prior to the official data collection, a pilot study was executed to assess the questionnaire’s content validity and reliability, yielding a Cronbach’s α of 0.814. The data collection occurred within vaccination clinics, facilitated by medical personnel with support from our research team. Informed written consent was obtained from each participant well in advance of data collection. Rigorous checks were performed on the completed questionnaires by investigators, ensuring completeness and accuracy. Participants were prompted to fill in any missing information to maintain the integrity of the collected data.

### Measures/questionnaire development

The questionnaire was developed with reference to previously published instruments assessing parental willingness to vaccinate for self-paid vaccines in China.^22,23^ The draft was reviewed by a panel of five public health experts and three clinicians to evaluate the clarity, relevance, and cultural appropriateness of each item. Following expert feedback, minor wording adjustments were made. A pilot test with 50 parents was conducted prior to the formal survey to examine reliability and content validity. The overall Cronbach’s α was 0.814, indicating satisfactory internal consistency, and the item-level content validity index (I-CVI) ranged from 0.83 to 1.00. Given that the questionnaire was primarily descriptive and scenario-based rather than a latent construct scale, exploratory or confirmatory factor analysis was not conducted.

The finalized questionnaire consisted of seven major sections: (1) Child demographics, including the number of children, sex, health status, and household registration; (2) Vaccination logistics and non-medical costs, covering travel distance, expenses, accompanying persons, and time spent; (3) Vaccination history and awareness, assessing knowledge of non-NIP vaccines, information sources, and overall attitudes; (4) COVID-19–related perceptions, capturing the pandemic’s influence on vaccine trust and willingness to pay; (5) Vaccine trust and decision factors, evaluating confidence in government and healthcare providers, price sensitivity, and service accessibility; (6) Parental sociodemographic characteristics, including education, occupation, income, religion, medical background, insurance type, and age. Together, these sections formed an integrated framework linking socioeconomic, cognitive, and behavioral factors to parents’ willingness to vaccinate their children against non-NIP diseases. (7) Willingness-to-pay and willingness-to-vaccinate under different reimbursement scenarios, measuring payment ceilings and 0–10 willingness scores across four non-NIP vaccines (pentavalent, varicella, EV71, influenza). Specifically, willingness to vaccinate (WTV) was assessed through two components. Participants first indicated the maximum amount they were willing to pay for the pentavalent vaccine (0–2396 CNY). Subsequently, they rated their vaccination willingness on a 0–10 scale (0 = very unwilling, 10 = very willing) under five hypothetical reimbursement scenarios (0%, 25%, 50%, 75%, and 100%). Higher scores indicated stronger willingness. The average rating across the five scenarios was calculated as each respondent’s overall WTV score for subsequent analyses.

The analysis included 31 covariates capturing parental, child, and contextual characteristics relevant to vaccination behavior, as described above. The outcome variable was the willingness-to-vaccinate (WTV) score, calculated as the mean of parents’ 0–10 ratings under five hypothetical reimbursement scenarios (0%, 25%, 50%, 75%, and 100%) and standardized to a 0–100 scale, with higher scores indicating greater willingness to vaccinate.

### Statistical analysis

All statistical analyses were performed in R (version 4.4.3). Descriptive statistics were used to summarize participants’ baseline characteristics and vaccination intentions, presented as frequencies and percentages for categorical variables and as means with standard deviations for continuous variables. Differences in categorical variables were tested using the chi-square test or Fisher’s exact test when appropriate, and differences in continuous variables were assessed using Wilcoxon rank sum test. A two-sided P value < 0.05 was considered statistically significant.

To examine factors associated with willingness to vaccinate, all 31 covariates introduced in Table 1 were simultaneously included in multiple logistic regression models based on theoretical relevance rather than univariate screening. This full-model approach avoided bias introduced by data-driven variable selection. Multicollinearity was evaluated using generalized variance inflation factors (GVIF). All variables had adjusted GVIF values < 2 (Supplemental Table S1), indicating no serious multicollinearity. Although parental occupation variables (mjob, fjob) showed slightly higher raw GVIFs due to multiple categorical levels, their adjusted GVIFs remained well below the conventional threshold of 5. To ensure model parsimony, these variables were excluded from the final model. To model repeated WTV scores across the five reimbursement scenarios, we fitted linear mixed-effects models with random effects at the individual level. To determine the optimal random-effect structure, three candidate models (random intercept only, random slope only, and random intercept + random slope) were compared using Akaike Information Criterion (AIC) and Bayesian Information Criterion (BIC). The comparison results have been added to the supplemental materials as Supplemental Table S2. The random intercept + random slope model yielded the lowest AIC/BIC and was therefore selected. The model used in current paper was specifically described as:

(1) $$y_{\mathrm{ij}}=\mathrm{alph}a_{i}+\mathrm{bet}a_{i}*\mathrm{rati}o_{\mathrm{ij}}+\mathrm{epsilo}n_{\mathrm{ij}},$$

where $y_{\mathrm{ij}}$ is the WTV score of the *i*th individual when reimbursement ratio level is *j* and $\mathrm{alph}a_{i}$ and $\mathrm{bet}a_{i}$ are the random intercept (baseline WTV) and random slope (change in WTV with reimbursement ratio), respectively, and are assumed to follow a bivariate normal distribution, with the within-subject correlation modeled under the symmetric correlation structure. $\mathrm{epsilo}n_{\mathrm{ij}}$ is the normally distributed measurement error. Linear mixed-effects models were fitted using the *lmer* function from the *lme4* package (Version 1.1.37) in R, which estimates both fixed and random effects to appropriately account for repeated measurements within individuals. Subject-specific predicted intercepts and slopes were then extracted from the fitted models for use in subsequent clustering analyses. The predicted random effects (intercepts and slopes) were used for unsupervised k-means clustering, using Euclidean distance and k-means++ initialization to ensure well-separated starting centers, thereby identifying subgroups of parents with distinct response patterns to reimbursement scenarios. Each participant’s random intercept (baseline WTV) and random slope (change in WTV with reimbursement ratio) were extracted from the mixed-effects model and standardized to form a two-dimensional feature space representing individual response profiles. The clustering analysis was performed using the R package *NbClust* (Version 3.0.1), which systematically evaluated solutions across a range of cluster numbers (k = 2–6). The optimal number of clusters was selected based on the majority consensus across internal validity indices, with each index applied according to its standard criterion, e.g., maximizing silhouette and Calinski – Harabasz (CH) indices, minimizing C-index, identifying local maxima for Krzanowski – Lai and Friedman-Rubin indices, and applying the one-standard-error rule for the Gap statistic (Supplemental table S3). The average silhouette coefficient for the chosen four-cluster solution was 0.6134, indicating good cluster separation and cohesion. Sociodemographic and behavioral characteristics were compared across clusters using chi-square tests for categorical variables and t-tests or ANOVA for continuous variables. Differences in mean WTV scores were further visualized to illustrate each subgroup’s willingness profile.

## Results

### Basic information of the study population

Table 1 shows the basic information of the study population. Univariate analysis revealed significant correlations between vaccine willingness and factors such as number of babies, age in months of baby, health condition of baby, household registration of baby, education of father, education of mother, family income, transport to vaccination, distance, vaccine known, number of information source, impacts of COVID-19 pandemic on parents’ perceptions of vaccines, vaccine fee, doctor recommendation, satisfaction of the set distance for vaccination clinics, satisfaction of operating hours, average waiting time and attitude of the staff.

**Table 1.** Basic characteristic of the study population stratified according to WTV (N = 1962).

Characteristic	Overall, N = 1962^a^	Unwilling, N = 171^a^	Willing, N = 1791^a^	*P*^b^	Characteristic	Overall, N = 1962^a^	Unwilling, N = 171^a^	Willing, N = 1791^a^	*P*^b^
**Basic information about children**					**Non-Medical Costs**				
Number of babies	2 (1)	2 (1)	2 (1)	.**025**	Transport to vaccination				**<.001**
Age in months of the baby	41 (32)	51 (32)	40 (31)	**<.001**	Walking	632 (32%)	76 (44%)	556 (31%)	
Gender of the baby				.800	Bicycle/e-bike	48 (2.4%)	7 (4.1%)	41 (2.3%)	
Male	1,027 (52%)	91 (53%)	936 (52%)		Private car	1,268 (65%)	86 (50%)	1,182 (66%)	
Female	935 (48%)	80 (47%)	855 (48%)		Bus/underground	12 (0.6%)	2 (1.2%)	10 (0.6%)	
Health condition of the baby				.**011**	Taxi/ride-hailing services	2 (0.1%)	0 (0%)	2 (0.1%)	
Healthy	1,838 (94%)	151 (88%)	1,687 (94%)		Distance to vaccination	2 (1)	2 (1)	2 (1)	**<.001**
General	115 (5.9%)	18 (11%)	97 (5.4%)		Travelling fees	2 (1)	2 (1)	2 (1)	.120
Poor	9 (0.5%)	2 (1.2%)	7 (0.4%)		Total time taken by parents	1 (1)	1 (0)	1 (1)	.086
Household registration of the baby				**<.001**	**Vaccination status**				
Urban	1,232 (63%)	87 (51%)	1,145 (64%)		Vaccine known	6 (4)	5 (3)	6 (4))	**<.001**
Rural	730 (37%)	84 (49%)	646 (36%)		**Awareness**				
Health insurance of the baby	1,852 (94%)	156 (91%)	1,696 (95%)	.060	Number of Information sources	2 (1)	2 (1)	2 (1)	.**007**
**Basic information about the parents**					Impacts of COVID-19 on the baby not timely vaccinations				.600
Age of father	34 (5)	35 (6)	34 (5)	.100	Yes	973 (50%)	88 (51%)	885 (49%)	
Job of father					No	989 (50%)	83 (49%)	906 (51%)	
Self-employed	649 (33%)	63 (37%)	586 (33%)		Impacts of COVID-19 on parents’ perceptions of vaccines				**<.001**
Farmers	68 (3.5%)	17 (9.9%)	51 (2.8%)		More trust	259 (13%)	17 (9.9%)	242 (14%)	
Medical staff	44 (2.2%)	6 (3.5%)	38 (2.1%)		More distrust	260 (13%)	43 (25%)	217 (12%)	
Teachers	49 (2.5%)	5 (2.9%)	44 (2.5%)		No effect	1,443 (74%)	111 (65%)	1,332 (74%)	
Civil servants	892 (45%)	57 (33%)	835 (47%)		**Vaccine Trust Situation**				
Others	245 (12%)	20 (12%)	225 (13%)		Impact of vaccine cost on WTV	2 (1)	2 (1)	2 (1)	**<.001**
No job	15 (0.8%)	3 (1.8%)	12 (0.7%)		Doctor recommendation	608 (31%)	37 (22%)	571 (32%)	.**006**
Education of father	2 (1)	2 (1)	3(1)	**<.001**	**Satisfaction**				
Age of mother	33 (5)	33 (5)	33(5)	.300	Satisfaction of the set distance for vaccination clinics				.**013**
Job of mother					Yes	1,841 (94%)	153 (89%)	1,688 (94%)	
Self-employed	426 (22%)	42 (25%)	384 (21%)		No	121 (6.2%)	18 (11%)	103 (5.8%)	
Farmers	57 (2.9%)	12 (7.0%)	45 (2.5%)		Satisfaction of opening hours of vaccination clinics				.**001**
Medical staff	123 (6.3%)	13 (7.6%)	110 (6.1%)		Yes	1,767 (90%)	142 (83%)	1,625 (91%)	
Teachers	105 (5.4%)	8 (4.7%)	97 (5.4%)		No	195 (9.9%)	29 (17%)	166 (9.3%)	
Civil servants	798 (41%)	49 (29%)	749 (42%)		Average waiting time	44 (31)	43 (31)	45 (31)	.500
Others	223 (11%)	19 (11%)	204 (11%)		Validity of official vaccination information				.**018**
No job	230 (12%)	28 (16%)	202 (11%)		Fully satisfied	546 (28%)	38 (22%)	508 (28%)	
Education of mother	3 (1)	2 (1)	3 (1)	**<.001**	Largely satisfied	923 (47%)	73 (43%)	850 (47%)	
Family income	3 (1)	3 (1)	3 (1)	**<.001**	Generally satisfied	437 (22%)	55 (32%)	382 (21%)	
Religious belief of family members				.800	Less satisfied	42 (2.1%)	3 (1.8%)	39 (2.2%)	
Buddhism	287 (15%)	22 (13%)	265 (15%)		Not satisfied at all	14 (0.7%)	2 (1.2%)	12 (0.7%)	
Christianity	135 (6.9%)	13 (7.6%)	122 (6.8%)		Satisfaction of the attitude of the staff				.**010**
No	1,540 (78%)	136 (80%)	1,404 (78%)		Very good	571 (29%)	35 (20%)	536 (30%)	
Family members of medical or vaccination workers				>.900	Good	1,039 (53%)	94 (55%)	945 (53%)	
Yes	288 (15%)	25 (15%)	263 (15%)		General	346 (18%)	41 (24%)	305 (17%)	
No	1,673 (85%)	146 (85%)	1,527 (85%)		Poor	4 (0.2%)	0 (0%)	4 (0.2%)	
Communicable diseases history of family members				.800	Very poor	2 (0.1%)	1 (0.6%)	1 (<0.1%)	
Viral hepatitis	1,560 (80%)	141 (82%)	1,419 (79%)						
Bacterial pneumonia	286 (15%)	22 (13%)	264 (15%)						
Chickenpox	92 (4.7%)	6 (3.5%)	86 (4.8%)						
Measles	18 (0.9%)	2 (1.2%)	16 (0.9%)						
No	6 (0.3%)	0 (0%)	6 (0.3%)						

^a^Mean (SD); n (%).

^b^Wilcoxon rank sum test; Pearson’s Chi-squared test; Fisher’s exact test.

### Association results by multiple logistic regression

In our study, we employed the multiple logistic regression model to highlight the significance of each variable (Table 2). The most crucial variables identified were *distance* (OR (95% CI): 0.68 (0.48–0.94), *P* = .021), *impact of vaccine cost* (OR (95% CI): 0.66(0.55–0.79), *P* < .001), *doctor recommendation* (OR (95% CI): 1.85(1.24–2.81), *P* = .003), *satisfaction of opening hours* (OR (95% CI):0.52(0.31–0.88), P = .013), and *known vaccine* (OR (95% CI): 1.11(1.05–1.18), *P* < .001).

**Table 2.** Association results of multiple logistic regression including all covariates in Table 1.

Variations	OR (95%CI)	*P*
Number of babies	0.80(0.57–1.13)	.207
Gender of the baby (reference: male)		
Female	1.05(0.75–1.48)	.770
Health condition of the baby	0.72(0.45–1.20)	.193
Household registration of the baby	0.80(0.54–1.19)	.271
Distance	0.68(0.48–0.94)	.**021** (*)
Travelling fees	0.99(0.83–1.19)	.945
Total time taken by parents	1.45(1.01–2.22)	.060
Number of Information sources	1.09(0.94–1.29)	.274
Impacts of COVID-19 on the baby not timely vaccinations (reference: yes)		
No	1.06(0.76–1.49)	.729
Impacts of COVID-19 on parents’ perceptions of vaccines	1.05(0.83–1.33)	.668
Impact of vaccine cost	0.66(0.55–0.79)	**<.001** (***)
Doctor recommendation	1.85(1.24–2.81)	.**003** (**)
Satisfaction of the distance (reference: yes)		
No	0.84(0.46–1.61)	.593
Satisfaction of opening hours (reference: yes)		
No	0.52(0.31–0.88)	.**013** (*)
transport to vaccination	1.16(0.97–1.38)	.105
Average waiting time	1.00(1.00–1.01)	.220
Validity of official vaccination information (reference: fully satisfied)		
Largely satisfied	0.89(0.56–1.39)	.611
Generally satisfied	0.79(0.46–1.37)	.407
Less satisfied	1.92(0.54–9.83)	.367
Not satisfied at all	0.60(0.13–4.49)	.560
Attitude of the staff	0.88(0.66–1.18)	.406
Job of father (reference: self-employed)		
Farmers	0.51(0.20–1.36)	.173
Medical staff	0.61(0.21–2.05)	.401
Teachers	0.65(0.23–2.16)	.440
Civil servants	1.22(0.77–1.93)	.402
Others	1.33(0.71–2.59)	.383
No job	0.72(0.18–3.86)	.671
Education of father	1.06(0.76–1.50)	.718
Job of mother (reference: self-employed)		
Farmers	1.03(0.35–3.19)	.954
Medical staff	0.85(0.35–2.15)	.733
Teachers	0.91(0.38–2.41)	.832
Civil servants	1.25(0.74–2.10)	.402
Others	1.13(0.57–2.30)	.731
No job	0.71(0.39–1.30)	.260
Education of mother	0.90(0.64–1.29)	.574
Family income	1.12(0.93–1.34)	.234
Religious belief of family members (reference: no)		
Buddhism	1.11(0.69–1.88)	.670
Christianity	1.01(0.55–2.03)	.970
Family members of medical or vaccination worker	1.22(0.64–2.20)	.521
Health insurance of the baby	1.24(0.63–2.30)	.518
Vaccine known	1.11(1.05–1.18)	**<.001** (***)
Communicable diseases history of family members	1.09(0.82–1.49)	.572
Age in months of the baby	0.99(0.99–1.00)	.**003** (**)
Age of father	0.97(0.92–1.02)	.243
Age of mother	1.05(0.99–1.12)	.126

Bold values indicate statistically significant results (*p* < .05). Asterisks denote significance levels: *p < .05, **p < .01, ***p < .001.

### Stratification of population by mixed-effects model and unsupervised clustering

**Figure 2.**
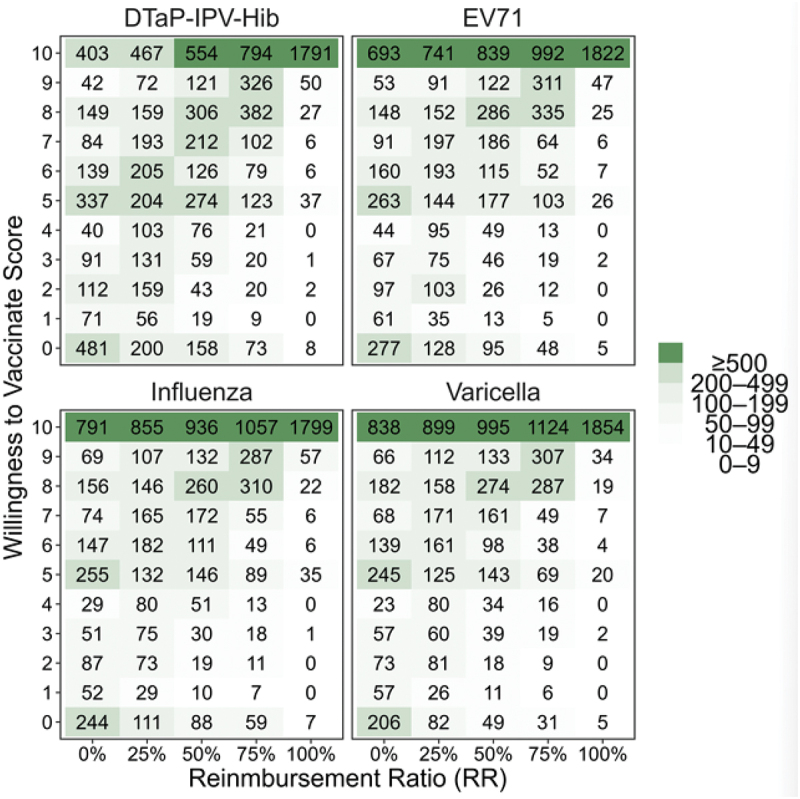
Distribution of willingness-to-vaccinate (WTV) scores across reimbursement ratios for the four self-paid vaccines. Each cell reports the mean WTV score at the corresponding reimbursement level. The color gradient reflects the underlying sample size contributing to each estimate: darker shades denote larger numbers of respondents, indicating more stable estimates, whereas lighter shades denote smaller subsamples with greater variability.

In our study, we implemented a linear mixed-effects model to explore the correlation between reimbursement proportion and WTV utilizing panel data collected at different reimbursement ratios. Figure 2 shows distributions of WTV scores for four self-paid vaccines. The color intensity in the graph reflects the sample counts within each reimbursement ratio and willingness-to-vaccinate score category, with darker shades indicating a larger number of people in that segment. Notably, the darker areas are predominantly clustered along the diagonal, aligning with the hypothesis that higher reimbursement ratios tend to correlate with increased willingness-to-vaccinate scores. We observed a noteworthy trend where a substantial proportion of survey participants indicated a high WTV, scoring 5 or above, regardless of lower financial incentives. This was particularly evident with the DTaP-IPV-Hib vaccine, where more than 65% of respondents reported a WTV with a score exceeding 5 points, even without any reimbursement offered. Such data underscore the varying degrees of cost-related responses across the population, indicating the population heterogeneity with respect to cost and vaccine willingness. Further removing samples with missing data resulted in the following sample sizes: 1949 for the DTaP-IPV-Hib vaccine, 1954 for the EV71 vaccine, 1955 for the Influenza vaccine, and 1955 for the Varicella vaccine.

We then utilized the k-means clustering algorithm, an unsupervised machine learning method, on scaled random effects, with the four-cluster solution selected as optimal through a consensus of internal validity indices (for K = 2–6) and visual inspection. Notably, the extent of change in WTV scores significantly differed among four unique categories: an extremely low willing and acute price-sensitive group (cluster 1), a low-willing and high price-sensitive group (cluster 2), a moderately willing and price-sensitive group (cluster 3), and a consistently high-willingness group (cluster 4).

**Figure 3.**
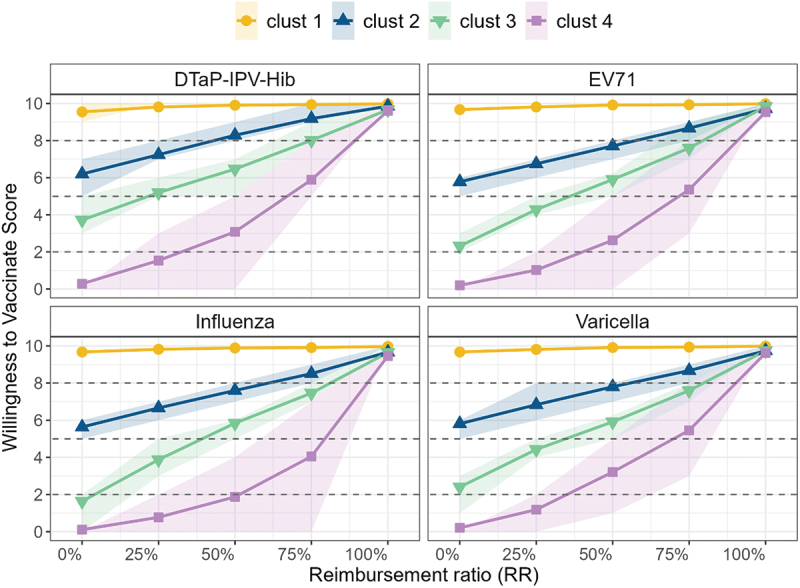
Average willingness-to-vaccinate (WTV) score curves across reimbursement ratios for the four parental clusters. Each line represents the mean WTV trajectory within a cluster, with shaded bands indicating 95% confidence intervals (CIs).

Figure 3 shows the average willingness-to-vaccinate (WTV) score trajectories for four different vaccines at varying reimbursement ratios. A key observation is the general trend of increasing WTV in response to rising ratios for each vaccine. Members of this subgroup show a reduced willingness to vaccinate and are more likely to bear the majority of vaccination costs through out-of-pocket payments. Furthermore, our analysis revealed vaccine-specific critical payment thresholds that significantly influence willingness levels. The consistently high willingness subgroup (cluster 4), as highlighted in Figure 3, manifests a pronounced inclination toward accepting self-paid vaccines, with a negligible response to variations in cost or reimbursement ratios. For instance, with the pentavalent vaccine, we observed that cluster 2 exhibited increased willingness when the reimbursement proportion exceeded 50%. In contrast, cluster 3 required a higher score, with increased willingness observed at reimbursement proportions above 75%. Meanwhile, cluster 1 exhibited a comparable increase in willingness only when the reimbursement rate surpassed 75%. The distribution of different cluster has been shown at Supplemental Table S5 and characteristic at Supplemental Table S6-S9. These findings highlight the nuanced relationship between payment proportions and willingness to vaccinate across different vaccines and population subgroups.

### Population characteristics of subgroups

**Figure 4.**
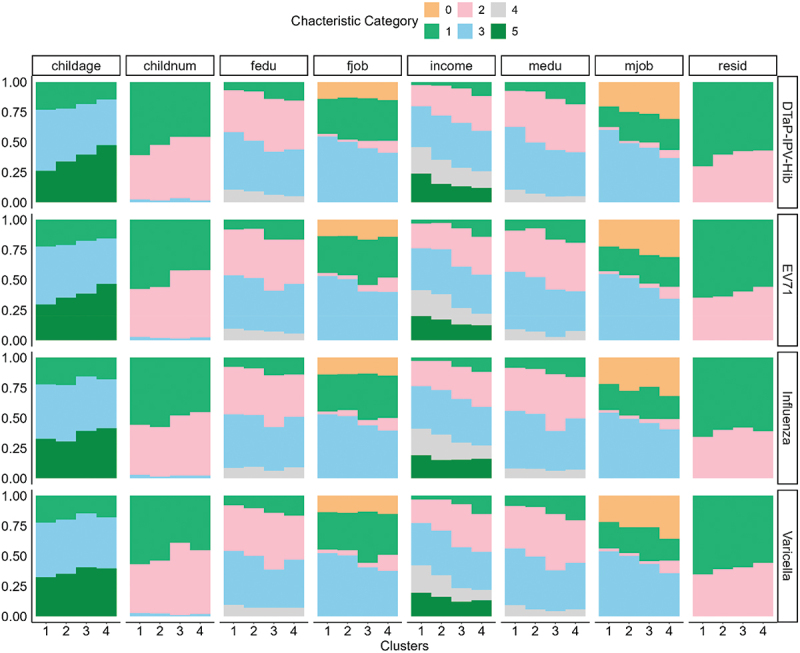
Population characteristics of subgroups. Subgroup characteristics comparison among four different clusters by unsupervised k-mean clustering on best linear unbiased predictors (BLUPs) from linear mixed effects model (LMM); **childage** (age group of child divided into): less than 1 y (1), 1~ 3 y (3), 3~5 y (5) groups; **childnum** (number of children in family): 1, 2, 3; **medu** and **fedu** (education levels of mother or father): bellow high school (1), high school (2), undergraduate (3) and graduate and above (4); **mjob** and **fjob** (job of mother or father): no job (0), other (1), farmer (2), medical related job or teacher or civil servant (3); **income**: below 5000yuan (1), 5000~1000yuan (2), 10,000~20,000yuan (3) 20000~30,000yuan (4) and 30000 above (5); **resid** (residence): urban (1) and rural area (2).

Figure 4 provides a comprehensive overview of the unique characteristics associated with each subgroup concerning the four self-administered vaccines. Across the four clusters, we observed two groups that were largely price-insensitive (Clusters 1 and 4) and two groups that showed clear price responsiveness (Clusters 2 and 3), possibly driven by distinct underlying mechanisms. Cluster 1, characterized by higher parental education and stronger health awareness, exhibited consistently high baseline willingness-to-vaccinate (WTV), resulting in minimal changes in WTV across reimbursement levels. Cluster 4 also showed weak price responsiveness, but this pattern reflected a markedly low baseline WTV: as a low-SES group with limited vaccine confidence and low perceived benefits, financial subsidies produced only minor increases in vaccination intention.

In contrast, Clusters 2 and 3 formed the price-sensitive group. Cluster 2, representing mid-SES families with moderate income and education, demonstrated substantial increases in WTV as reimbursement rose, consistent with cost – benefit considerations in households that must balance medical expenses within their budgets. Cluster 3 displayed the highest degree of price responsiveness. As a relatively low-SES group compared with Cluster 2, they faced clearer ability-to-pay constraints. Even small out-of-pocket costs could suppress WTV, making reimbursement particularly effective in increasing their likelihood of vaccination. Together, these results indicate that price sensitivity is driven by budgetary trade-offs among mid-SES families (Cluster 2) and financial constraints among low-SES families (Cluster 3), whereas price insensitivity reflects either high intrinsic vaccination intent (Cluster 1) or structural hesitancy (Cluster 4).

## Discussion

This study employed a mixed-effects model and was based on a survey of 2212 households in Hangzhou, Zhejiang Province, to explore the willingness to vaccinate (WTV) for four different types of self-paid childhood vaccines. Our study provides several novel insights into the determinants of WTV for self-paid vaccines among Chinese parents, particularly in the context of different reimbursement ratios. The key findings highlight that vaccination cost is a critical barrier to WTV, and we identified distinct population subgroups with varying levels of cost sensitivity and willingness to vaccinate. These findings have important implications for vaccination policies and strategies aimed at improving vaccine uptake.

Our study utilized a multiple logistic regression model to identify the most crucial variables influencing WTV. The results revealed that distance, impact of vaccine cost, doctor recommendation, satisfaction with vaccination clinic operating hours, and vaccine knowledge were the most significant factors. These findings align with and extend previous research on vaccine hesitancy and cost sensitivity. For instance, studies on influenza and pneumococcal vaccines have consistently identified cost as a significant barrier to vaccination, particularly in low- and middle-income settings.^14,20^ The impact of vaccine cost was particularly pronounced, with higher reimbursement ratios associated with increased WTV scores. This was especially evident for the DTaP-IPV-Hib vaccine, where the proportion of respondents with a WTV score above 5 increased from 58.9% to 99.5%. However, for other vaccines like EV71, influenza, and varicella, a substantial proportion of participants indicated high WTV regardless of financial incentives. The lack of public awareness, coupled with the high price of combination vaccines, may require more substantial financial incentives to achieve similar levels of acceptance, as confirmed by other researchers.^24–26^ This underscores the need for vaccine-specific strategies in policy design, as different vaccines may require varying levels of financial incentives to achieve similar acceptance rates.

In addition to cost, doctor recommendations emerged as a critical factor influencing WTV. This finding is consistent with previous research showing that healthcare professionals play a pivotal role in shaping parental attitudes toward vaccination.^12,25,27^ Furthermore, satisfaction with vaccination clinic operating hours was also a significant predictor of WTV, highlighting the importance of accessibility and convenience in vaccination programs. These findings suggest that improving healthcare provider communication and optimizing clinic operations could enhance vaccine uptake.

To our knowledge, this study represents a pioneering investigation into the interplay between vaccination reimbursement rates and parental willingness scores within the context of voluntary pediatric immunization. The analysis uncovered substantial heterogeneity in cost sensitivity across socioeconomic strata, with unsupervised clustering delineating four distinct behavioral archetypes: an extremely low willing and acute price-sensitive group, a low-willing and high price-sensitive group, a moderately willing and price-sensitive group, and a consistently high-willingness group. Notably, families characterized by higher socioeconomic status, fewer children, and higher parental educational attainment maintained strong WTV regardless of reimbursement levels. This finding aligns with the researches showing that higher education and income levels are associated with greater vaccine acceptance.^11,28^ Conversely, economically disadvantaged households with less educated parents exhibited pronounced cost sensitivity, revealing critical equity gaps in immunization access. Similar to prior reports of structural vaccination barriers among highly educated populations,^29,30^ our data localize such impediments specifically to parents with intermediate (non-advanced) education levels, whose healthcare expenditure patterns reflect either risk-aversion behaviors or budget prioritization dilemmas. It is likely that parental concerns regarding vaccine efficacy have impacted vaccination willingness, as such caregivers often possess an incomplete understanding of immunization information yet maintain heightened vigilance. There was also one recent study that implied that prenatal capacity to critically analyze vaccine-related crisis information exhibited a positive correlation with both immunization willingness.^31^

In our analysis, neither fathers’ nor mothers’ educational levels, nor their interaction, showed a significant association with WTV. This suggests that parental education may not directly shape vaccine willingness in this cohort, potentially reflecting high baseline awareness and access to vaccination information. Cultural context may still be relevant: fathers often take the lead in economic decision-making, and lower paternal education may heighten cost sensitivity, while mothers commonly guide health information seeking and preventive behaviors. Although maternal education did not moderate the association, maternal health literacy may still influence vaccination decisions. Larger and more diverse samples are needed to clarify these within-family dynamics. The socioeconomic context of Zhejiang may also contribute to the null interaction. As a highly developed province, household income and vaccine affordability are relatively high, reducing variability in perceived financial burden across families. This economic homogeneity may attenuate any moderating effect of maternal education. Studies including regions with broader socioeconomic variation may help clarify how educational and financial roles jointly influence vaccine decision-making.

Our findings indicate substantial heterogeneity in willingness-to-vaccinate across the four self-paid vaccines, and this variation appears to reflect parents’ implicit evaluations of vaccine benefits relative to costs. The DTaP-IPV/Hib vaccine – despite its higher nominal price – demonstrated the strongest baseline acceptance and required the lowest reimbursement threshold. This pattern is consistent with its broad protection against five historically severe childhood diseases, which parents may perceive as offering a favorable “value-for-money” profile. In contrast, EV71, influenza, and varicella vaccines target conditions that, while highly prevalent, generally have lower fatality rates and fewer long-term sequelae in contemporary China, resulting in a higher reimbursement requirement to reach comparable acceptance levels. By integrating vaccine price, disease burden, and perceived cost-effectiveness, the observed WTV differences reflect a rational, benefit-driven decision-making process rather than price sensitivity alone. This interpretation is further supported by the comparative vaccine characteristics summarized in Supplemental TableS4, which highlights that both economic considerations and perceived health benefits jointly shape parental vaccination preferences.

## Limitations and strengths

Our study stands out methodologically by combining machine learning techniques with linear mixed-effects modeling to analyze data on vaccination willingness. This approach allowed us to capture both individual-level variability and population-level trends, providing a more nuanced understanding of how reimbursement ratios influence vaccination willingness. While previous studies have often focused on single type of vaccine, our research examined four different self-paid vaccines (EV71, varicella, influenza, and DTaP-IPV-Hib), offering a broader perspective on vaccine-specific cost sensitivities. Additionally, our data were collected through structured questionnaires, ensuring high-quality, first-hand information from a diverse sample of households in Hangzhou, Zhejiang Province, and comprehensively evaluating the economic and psychosocial factors influencing vaccination willingness.

Several limitations should be acknowledged. First, this study was conducted in Hangzhou, an economically developed city in eastern China, and the findings may not be directly generalizable to regions with different socioeconomic and healthcare contexts. Regional variation in immunization delivery and financing for self-paid vaccines in China may lead to different levels of cost sensitivity, and our findings should therefore be interpreted in the local context and validated in multi-region samples. Although a stratified sampling design across three districts helped capture within-city heterogeneity, external representativeness remains limited. Second, publicly available data on self-paid (non-NIP) childhood vaccine coverage at the city, provincial, or national levels are currently lacking, which precluded direct benchmarking of our sample against broader reference data. Third, willingness-to-vaccinate (WTV) was self-reported and may be subject to social desirability bias. Although anchor questions on previous self-paid vaccination behavior were included, future longitudinal designs linking WTV with actual vaccination records would help validate behavioral consistency. Fourth, several potentially important factors, such as parental perceptions of vaccine safety, trust in healthcare providers, and service-quality dimensions beyond opening hours (for example waiting time or staff communication) were not measured and may introduce residual confounding. Incorporating validated vaccine hesitancy and service-quality scales in future work would strengthen model completeness. Finally, the four WTV subgroups identified through clustering were derived from a single dataset without external validation. Replication in independent cohorts or multi-city samples is required to confirm their stability and generalizability.

Despite these limitations, this study provides one of the first large-scale, multi-vaccine assessments of parental cost sensitivity and willingness-to-vaccinate in China, offering an empirical basis for policy discussions on reimbursement strategies. As a pilot study, the present work primarily aimed to test data quality, analytic feasibility, and methodological pathways for assessing self-paid vaccine coverage using the Zhejiang Immunization Information System. We observed heterogeneity across clusters in responsiveness to reimbursement, supporting a tiered approach. As scenario-based examples, the most responsive cluster could be prioritized for ≥75% reimbursement, whereas a ≥ 50% reimbursement for DTaP-IPV-Hib may be sufficient in a moderately responsive cluster; exact thresholds should be locally calibrated based on budget impact and baseline coverage. Considering budget constraints, subsidies could be prioritized by vaccine type, focusing first on higher-cost vaccines that show stronger responsiveness to reimbursement. For example, DTaP-IPV-Hib may merit earlier or higher subsidy, whereas lower-cost vaccines may achieve gains with smaller reimbursement tiers. Building upon these experiences, future multicenter studies could extend this framework to provinces representing eastern, central, and western China, where socioeconomic and healthcare contexts differ markedly. For the future, we propose a stepwise implementation: first pilot the reimbursement strategy in selected areas, then scale up if predefined targets are met. As practical targets, programs could aim for a ≥ 10–15% increase in vaccine uptake from the local baseline within 6–12 months and continue monitoring coverage and equity during scale-up. Such coordinated studies would enable cross-regional comparisons, promote harmonization of provincial immunization information systems, and provide evidence for optimizing strategies to improve vaccine accessibility and equity nationwide.

## Conclusions

In conclusion, our study employed logistic regression to examine the determinants of self-paid vaccination willingness and built a mixed-effects model to stratify populations into subgroups based on price sensitivity and vaccination willingness. The findings underscore that the impact of cost is the predominant obstacle to vaccination willingness. Families with fewer children, younger children, higher-educated parents, and those of higher socioeconomic status consistently display strong intentions to vaccinate. In addition, the educational level of the father emerges as a key indicator of sensitivity to vaccination costs.

## Supplementary Material

Supplemental MaterialsR2submit_clean.docx

## Data Availability

Data are available upon reasonable request.
