# Supplementary material for: Exploring the impact of reimbursement ratios on willingness to vaccinate: A mixed-effects modeling approach using panel data
Source: Hum Vaccin Immunother. 2026 Mar 3;22(1):2609339. doi: 10.1080/21645515.2025.2609339 (PMC12959184; doi:10.1080/21645515.2025.2609339)
Supplement: Supplemental MaterialsR2submit_clean.docx [file KHVI_A_2609339_SM7400.docx]

**Supplemental Materials**

**Supplemental Table S1.** Generalized Variance Inflation Factors (GVIFs) used for collinearity diagnostics of candidate predictors in multivariable logistic regression models

|  | **GVIF** | **Df** | **GVIF^(1/(2*Df))** |
| --- | --- | --- | --- |
| Number of babies | 1.32 | 1 | 1.15 |
| Gender of the baby | 1.05 | 1 | 1.03 |
| Health condition of the baby | 1.09 | 1 | 1.04 |
| Household registration of the baby | 1.43 | 1 | 1.20 |
| Distance | 1.22 | 1 | 1.10 |
| Travelling fees | 1.22 | 1 | 1.10 |
| Total time taken by parents | 1.05 | 1 | 1.02 |
| Number of Information sources | 1.09 | 1 | 1.05 |
| Impacts of COVID-19 on the baby not timely vaccinations | 1.06 | 2 | 1.01 |
| Impacts of COVID-19 on parents' perceptions of vaccines | 1.07 | 1 | 1.03 |
| Impact of vaccine cost | 1.15 | 1 | 1.07 |
| Doctor recommendation | 1.06 | 1 | 1.03 |
| Satisfaction of the distance | 1.21 | 1 | 1.10 |
| Satisfaction of opening hours | 1.95 | 2 | 1.18 |
| transport to vaccination | 1.93 | 4 | 1.09 |
| Average waiting time | 1.11 | 1 | 1.05 |
| Validity of official vaccination information | 1.45 | 1 | 1.21 |
| Attitude of the staff | 1.45 | 1 | 1.20 |
| Job of father | 8.26 | 6 | 1.19 |
| Education of father | 2.78 | 1 | 1.67 |
| Job of mother | 10.39 | 6 | 1.22 |
| Education of mother | 2.64 | 1 | 1.62 |
| Family income | 1.40 | 1 | 1.18 |
| Religious belief of family members | 1.13 | 4 | 1.01 |
| Family members of medical or vaccination worker | 1.73 | 1 | 1.31 |
| Health insurance of the baby | 1.12 | 1 | 1.06 |
| Vaccine known | 1.16 | 1 | 1.08 |
| Communicable diseases history of family members | 1.10 | 1 | 1.05 |
| Age in months of the baby | 1.45 | 1 | 1.20 |
| Age of father | 3.58 | 1 | 1.89 |
| Age of mother | 3.75 | 1 | 1.94 |

**Supplemental Table S2.** Model fit comparison for random-effects structures

| **Vaccine** | **Model^1^** | **AIC** | **BIC** |
| --- | --- | --- | --- |
| DTaP-IPV-Hib | rand_int_slope | **43031.32** | **43075.05** |
| DTaP-IPV-Hib | rand_intercept | 48144.84 | 48173.99 |
| DTaP-IPV-Hib | rand_slope | 53474.75 | 53503.90 |
| Varicella | rand_int_slope | **37793.84** | **37837.55** |
| Varicella | rand_intercept | 45137.28 | 45166.42 |
| Varicella | rand_slope | 50716.9 | 50746.04 |
| EV71 | rand_int_slope | **39897.68** | **39941.40** |
| EV71 | rand_intercept | 46625.15 | 46654.30 |
| EV71 | rand_slope | 52307.27 | 52336.42 |
| Influenza | rand_int_slope | **39470.31** | **39514.03** |
| Influenza | rand_intercept | 45817.64 | 45846.78 |
| Influenza | rand_slope | 51932.38 | 51961.52 |
| ^1^rand_int_slope: model includes both random intercept and slope; rand_intercept: model includes only random intercept; rand_slope: model includes only random slope. | | | |

**Supplemental Table S3**:Internal validity indices used to identify the optimal number of clusters (K = 2–6). For each index, the value corresponding to the optimal cluster number is highlighted. The optimal K was selected based on majority consensus across indices.

| Index | Number of Clusters | Index Value | Optimal K |
| --- | --- | --- | --- |
| Calinski–Harabasz (CH) Index | 2 | 6147.882 |  |
| Calinski–Harabasz (CH) Index | 3 | 10817.9619 |  |
| **Calinski–Harabasz (CH) Index** | **4** | **11997.6281** | ✓ |
| Calinski–Harabasz (CH) Index | 5 | 11080.226 |  |
| Calinski–Harabasz (CH) Index | 6 | 9366.2371 |  |
| Gap | 2 | -0.1075 |  |
| **Gap** | **3** | **0.0995** | ✓ |
| Gap | 4 | 0.0083 |  |
| Gap | 5 | -0.2386 |  |
| Gap | 6 | -0.5414 |  |
| C-Index | 2 | 0.3625 |  |
| C-Index | 3 | 0.2753 |  |
| **C-Index** | **4** | **0.2437** | ✓ |
| C-Index | 5 | 0.2458 |  |
| C-Index | 6 | 0.2533 |  |
| Silhouette Coefficient | 2 | 0.6255 |  |
| **Silhouette Coefficient** | **3** | **0.6528** | ✓ |
| Silhouette Coefficient | 4 | 0.6134 |  |
| Silhouette Coefficient | 5 | 0.5682 |  |
| Silhouette Coefficient | 6 | 0.5424 |  |
| Krzanowski–Lai (KL) Index | 2 | 1.9275 |  |
| Krzanowski–Lai (KL) Index | 3 | 5.3898 |  |
| **Krzanowski–Lai (KL) Index** | **4** | **7.9213** | ✓ |
| Krzanowski–Lai (KL) Index | 5 | 0.181 |  |
| Krzanowski–Lai (KL) Index | 6 | 0.5723 |  |
| Friedman–Rubin (Rubin) Index | 2 | 3.8357 |  |
| Friedman–Rubin (Rubin) Index | 3 | 10.9843 |  |
| **Friedman–Rubin (Rubin) Index** | **4** | **22.641** | ✓ |
| Friedman–Rubin (Rubin) Index | 5 | 21.4715 |  |
| Friedman–Rubin (Rubin) Index | 6 | 17.6172 |  |

**Supplemental Table S4.** Comparative characteristics of the four vaccines, including single-dose price, recommended number of doses, out-of-pocket cost under different reimbursement levels (0%, 50%, 75%), disease incidence and mortality, perceived severity, and vaccine effectiveness. These data are provided to support the mechanistic interpretation of vaccine-specific differences in willingness-to-vaccinate (WTV) and reimbursement sensitivity. OOP per dose = price × (1 – reimbursement).

| **Vaccine** | **Single-dose price (RMB)** | **Recommended doses** | **OOP per dose**  **(0% / 50% / 75%)** | **Target diseases** | **Disease burden in China (incidence & mortality)** | **Notes on perceived severity / effectiveness** |
| --- | --- | --- | --- | --- | --- | --- |
| **EV71** | 188 | 2 | 188 / 94 / 47 | Severe HFMD caused by EV-A71 | • HFMD median incidence ~153.8 /100,000 (2011–18)  • CFR ~0.03%^1^  • 28.3% drop in incidence and 83.8% drop in mortality after EV71 vaccine introduction^2^ | EV71 targets the *severe* form of HFMD; overall HFMD mortality is low in recent years |
| **Influenza** | 128 | 1 | 128 / 64 / 32 | Seasonal influenza | • National influenza-associated excess mortality: ~14.5 /100,000  • ~88,100 excess respiratory deaths annually | Disease is common but severity varies; perceived as seasonal & self-limiting by many parents |
| **Varicella** | 136 | 2 | 136 / 68 / 34 | Chickenpox | • Incidence ~55–1270 /100,000 depending on source  • DALY burden declined from ~17.7 to ~4.7 (1990–2021)  • Mortality extremely low | Highly contagious but low mortality; perception of “mild childhood illness” reduces urgency |
| **DTaP-IPV/Hib** | 599 | 4 | 599 / 300 / 150 | Diphtheria, tetanus, pertussis, polio, Hib pneumonia/meningitis | • Historically high mortality before vaccination programs^3^  • Diphtheria essentially eliminated since 2006^4^  • Pertussis resurgence: 487,658 cases & 28 deaths in 2024^5^ | Protects against 5 severe diseases; high perceived benefit and strong parental risk aversion |

**References**

1. Xing W, Liao Q, Viboud C, et al. Hand, foot, and mouth disease in China, 2008&#x2013;12: an epidemiological study. *The Lancet Infectious Diseases*. 2014;14(4):308-318. doi:10.1016/S1473-3099(13)70342-6

2. Hong J, Liu F, Qi H, et al. Changing epidemiology of hand, foot, and mouth disease in China, 2013&#x2212;2019: a population-based study. *The Lancet Regional Health – Western Pacific*. 2022;20doi:10.1016/j.lanwpc.2021.100370

3. Yu W, Lee LA, Liu Y, et al. Vaccine-preventable disease control in the People's Republic of China: 1949-2016. *Vaccine*. Dec 18 2018;36(52):8131-8137. doi:10.1016/j.vaccine.2018.10.005

4. Yang Z, Feng T, Guan W, et al. Chinese expert consensus on immunoprophylaxis of common respiratory pathogens in children (2021 edition). *J Thorac Dis*. Mar 2022;14(3):749-768. doi:10.21037/jtd-21-1613

5. Zhang S, Liu J. Optimization of Diphtheria, Tetanus and Pertussis (DTP) vaccination strategy in China. *Journal of Infection*. 2025;90(2)doi:10.1016/j.jinf.2025.106416

**Supplemental Table S5.** Distribution of Willingness-to-Vaccinate (WTV) scores by vaccine type and reimbursement ratio (RR)

| **Vaccine** | **Reimbursement Ratio, RR** | **Mean ± SD** |
| --- | --- | --- |
| DTaP-IPV-Hib | 0% | 4.82 ± 3.73 |
| DTaP-IPV-Hib | 25% | 5.83 ± 3.35 |
| DTaP-IPV-Hib | 50% | 6.81 ± 3.05 |
| DTaP-IPV-Hib | 75% | 8.17 ± 2.44 |
| DTaP-IPV-Hib | 100% | 9.77 ± 1.05 |
| EV71 | 0% | 6.15 ± 3.68 |
| EV71 | 25% | 6.93 ± 3.21 |
| EV71 | 50% | 7.70 ± 2.78 |
| EV71 | 75% | 8.60 ± 2.17 |
| EV71 | 100% | 9.83 ± 0.88 |
| Influenza | 0% | 6.55 ± 3.64 |
| Influenza | 25% | 7.32 ± 3.12 |
| Influenza | 50% | 7.96 ± 2.69 |
| Influenza | 75% | 8.64 ± 2.26 |
| Influenza | 100% | 9.80 ± 0.98 |
| Varicella | 0% | 6.79 ± 3.55 |
| Varicella | 25% | 7.54 ± 2.99 |
| Varicella | 50% | 8.22 ± 2.45 |
| Varicella | 75% | 8.88 ± 1.95 |
| Varicella | 100% | 9.86 ± 0.82 |

**Supplemental Table S6.** Cluster-specific sociodemographic and behavioural characteristics for the DTaP-IPV-Hib vaccine

| **Characteristic** | **Cluster 1**  N = 542^1^ | **Cluster 2**  N = 380^1^ | **Cluster 3**  N = 470^1^ | **Cluster 4**  N = 557^1^ | **p**^2^ |
| --- | --- | --- | --- | --- | --- |
| **Number of babies** | 1 ± 1 | 1 ± 1 | 2 ± 1 | 2 ± 1 | <0.001 |
| **Gender of the baby** |  |  |  |  | 0.3 |
| Male | 272 (50%) | 215 (57%) | 241 (51%) | 295 (53%) |  |
| Female | 270 (50%) | 165 (43%) | 229 (49%) | 262 (47%) |  |
| **Health condition of the baby** |  |  |  |  | 0.016 |
| Healthy | 517 (95%) | 359 (94%) | 441 (94%) | 510 (92%) |  |
| General | 25 (4.6%) | 21 (5.5%) | 27 (5.7%) | 40 (7.2%) |  |
| Poor | 0 (0%) | 0 (0%) | 2 (0.4%) | 7 (1.3%) |  |
| **Household registration of the baby** |  |  |  |  | <0.001 |
| Urban | 377 (70%) | 239 (63%) | 282 (60%) | 325 (58%) |  |
| Rural | 165 (30%) | 141 (37%) | 188 (40%) | 232 (42%) |  |
| **Job of father** |  |  |  |  | <0.001 |
| no job | 71 (13%) | 55 (14%) | 47 (10%) | 85 (15%) |  |
| others | 161 (30%) | 119 (31%) | 181 (39%) | 180 (32%) |  |
| farmer | 12 (2.2%) | 4 (1.1%) | 11 (2.3%) | 41 (7.4%) |  |
| medical related job or teacher or civil servant | 298 (55%) | 202 (53%) | 231 (49%) | 251 (45%) |  |
| **Education of father** | 3 ± 1 | 3 ± 1 | 2 ± 1 | 2 ± 1 | <0.001 |
| **Job of mother** |  |  |  |  | <0.001 |
| no job | 110 (20%) | 79 (21%) | 115 (24%) | 146 (26%) |  |
| others | 103 (19%) | 75 (20%) | 116 (25%) | 128 (23%) |  |
| farmer | 11 (2.0%) | 7 (1.8%) | 7 (1.5%) | 32 (5.7%) |  |
| medical related job or teacher or civil servant | 318 (59%) | 219 (58%) | 232 (49%) | 251 (45%) |  |
| **Education of mother** | 3 ± 1 | 3 ± 1 | 2 ± 1 | 2 ± 1 | <0.001 |
| **Family income** | 3 ± 1 | 3 ± 1 | 3 ± 1 | 3 ± 1 | <0.001 |
| **Religious belief of family members** |  |  |  |  | 0.9 |
| None religous | 423 (78%) | 294 (77%) | 373 (79%) | 441 (79%) |  |
| Religous | 119 (22%) | 86 (23%) | 97 (21%) | 116 (21%) |  |
| **Age in months of the baby** | 34 ± 30 | 37 ± 31 | 41 ± 32 | 48 ± 31 | <0.001 |
| **Age of father** | 34 ± 5 | 34 ± 5 | 34 ± 5 | 35 ± 5 | <0.001 |
| **Age of mother** | 33 ± 4 | 33 ± 5 | 33 ± 5 | 33 ± 5 | 0.010 |
| ^1^Mean ± SD; n (%) | | | | | |
| ^2^Kruskal-Wallis rank sum test; Pearson's Chi-squared test; Fisher's exact test | | | | | |

**Supplemental Table S7.** Cluster-specific sociodemographic and behavioural characteristics for the EV71 vaccine

| **Characteristic** | **Cluster 1**  N = 853^1^ | **Cluster 2**  N = 555^1^ | **Cluster 3**  N = 268^1^ | **Cluster 4**  N = 278^1^ | **p**^2^ |
| --- | --- | --- | --- | --- | --- |
| **Number of babies** | 1 ± 1 | 1 ± 1 | 2 ± 1 | 2 ± 1 | <0.001 |
| **Gender of the baby** |  |  |  |  | 0.6 |
| Male | 447 (52%) | 282 (51%) | 139 (52%) | 155 (56%) |  |
| Female | 406 (48%) | 273 (49%) | 129 (48%) | 123 (44%) |  |
| **Health condition of the baby** |  |  |  |  | 0.008 |
| Healthy | 813 (95%) | 518 (93%) | 243 (91%) | 256 (92%) |  |
| General | 40 (4.7%) | 34 (6.1%) | 22 (8.2%) | 19 (6.8%) |  |
| Poor | 0 (0%) | 3 (0.5%) | 3 (1.1%) | 3 (1.1%) |  |
| **Household registration of the baby** |  |  |  |  | 0.084 |
| Urban | 552 (65%) | 348 (63%) | 169 (63%) | 156 (56%) |  |
| Rural | 301 (35%) | 207 (37%) | 99 (37%) | 122 (44%) |  |
| **Job of father** |  |  |  |  | <0.001 |
| no job | 105 (12%) | 73 (13%) | 39 (15%) | 41 (15%) |  |
| others | 268 (31%) | 179 (32%) | 104 (39%) | 94 (34%) |  |
| farmer | 19 (2.2%) | 13 (2.3%) | 10 (3.7%) | 26 (9.4%) |  |
| medical related job or teacher or civil servant | 461 (54%) | 290 (52%) | 115 (43%) | 117 (42%) |  |
| **Education of father** | 3 ± 1 | 3 ± 1 | 2 ± 1 | 2 ± 1 | <0.001 |
| **Job of mother** |  |  |  |  | <0.001 |
| no job | 179 (21%) | 126 (23%) | 67 (25%) | 79 (28%) |  |
| others | 175 (21%) | 115 (21%) | 66 (25%) | 67 (24%) |  |
| farmer | 17 (2.0%) | 11 (2.0%) | 7 (2.6%) | 22 (7.9%) |  |
| medical related job or teacher or civil servant | 482 (57%) | 303 (55%) | 128 (48%) | 110 (40%) |  |
| **Education of mother** | 3 ± 1 | 3 ± 1 | 2 ± 1 | 2 ± 1 | <0.001 |
| **Family income** | 3 ± 1 | 3 ± 1 | 3 ± 1 | 3 ± 1 | <0.001 |
| **Religious belief of family members** |  |  |  |  | 0.9 |
| None religous | 664 (78%) | 438 (79%) | 211 (79%) | 222 (80%) |  |
| Religous | 189 (22%) | 117 (21%) | 57 (21%) | 56 (20%) |  |
| **Age in months of the baby** | 38 ± 32 | 40 ± 31 | 44 ± 28 | 48 ± 33 | <0.001 |
| **Age of father** | 34 ± 5 | 34 ± 5 | 35 ± 6 | 36 ± 5 | <0.001 |
| **Age of mother** | 33 ± 5 | 33 ± 5 | 33 ± 5 | 34 ± 5 | 0.001 |
| ^1^Mean ± SD; n (%) | | | | | |
| ^2^Kruskal-Wallis rank sum test; Pearson's Chi-squared test; Fisher's exact test | | | | | |

**Supplemental Table S8.** Cluster-specific sociodemographic and behavioural characteristics for the varicella vaccine

| **Characteristic** | **Cluster 1**  N = 1035^1^ | **Cluster 2**  N = 488^1^ | **Cluster 3**  N = 228^1^ | **Cluster 4**  N = 204^1^ | **p**^2^ |
| --- | --- | --- | --- | --- | --- |
| **Number of babies** | 1 ± 1 | 1 ± 1 | 2 ± 1 | 2 ± 1 | <0.001 |
| **Gender of the baby** |  |  |  |  | 0.2 |
| Male | 538 (52%) | 247 (51%) | 118 (52%) | 121 (59%) |  |
| Female | 497 (48%) | 241 (49%) | 110 (48%) | 83 (41%) |  |
| **Health condition of the baby** |  |  |  |  | 0.031 |
| Healthy | 977 (94%) | 457 (94%) | 207 (91%) | 190 (93%) |  |
| General | 57 (5.5%) | 29 (5.9%) | 18 (7.9%) | 11 (5.4%) |  |
| Poor | 1 (<0.1%) | 2 (0.4%) | 3 (1.3%) | 3 (1.5%) |  |
| **Household registration of the baby** |  |  |  |  | 0.2 |
| Urban | 671 (65%) | 298 (61%) | 139 (61%) | 118 (58%) |  |
| Rural | 364 (35%) | 190 (39%) | 89 (39%) | 86 (42%) |  |
| **Job of father** |  |  |  |  | <0.001 |
| no job | 128 (12%) | 66 (14%) | 28 (12%) | 36 (18%) |  |
| others | 331 (32%) | 154 (32%) | 95 (42%) | 66 (32%) |  |
| farmer | 23 (2.2%) | 16 (3.3%) | 8 (3.5%) | 21 (10%) |  |
| medical related job or teacher or civil servant | 553 (53%) | 252 (52%) | 97 (43%) | 81 (40%) |  |
| **Education of father** | 3 ± 1 | 2 ± 1 | 2 ± 1 | 2 ± 1 | 0.002 |
| **Job of mother** |  |  |  |  | <0.001 |
| no job | 213 (21%) | 117 (24%) | 53 (23%) | 68 (33%) |  |
| others | 220 (21%) | 104 (21%) | 61 (27%) | 39 (19%) |  |
| farmer | 25 (2.4%) | 8 (1.6%) | 7 (3.1%) | 17 (8.3%) |  |
| medical related job or teacher or civil servant | 577 (56%) | 259 (53%) | 107 (47%) | 80 (39%) |  |
| **Education of mother** | 3 ± 1 | 2 ± 1 | 2 ± 1 | 2 ± 1 | <0.001 |
| **Family income** | 3 ± 1 | 3 ± 1 | 3 ± 1 | 3 ± 1 | <0.001 |
| **Religious belief of family members** |  |  |  |  | 0.2 |
| None religous | 799 (77%) | 381 (78%) | 187 (82%) | 169 (83%) |  |
| Religous | 236 (23%) | 107 (22%) | 41 (18%) | 35 (17%) |  |
| **Age in months of the baby** | 39 ± 32 | 40 ± 30 | 45 ± 29 | 44 ± 34 | 0.008 |
| **Age of father** | 34 ± 5 | 34 ± 5 | 35 ± 5 | 35 ± 5 | 0.018 |
| **Age of mother** | 33 ± 5 | 33 ± 5 | 34 ± 5 | 33 ± 5 | 0.018 |
| ^1^Mean ± SD; n (%) | | | | | |
| ^2^Kruskal-Wallis rank sum test; Pearson's Chi-squared test; Fisher's exact test | | | | | |

**Supplemental Table S9.** Cluster-specific sociodemographic and behavioural characteristics for the Influenza vaccine

| **Characteristic** | **Cluster 1**  N = 978^1^ | **Cluster 2**  N = 547^1^ | **Cluster 3**  N = 236^1^ | **Cluster 4**  N = 194^1^ | **p**^2^ |
| --- | --- | --- | --- | --- | --- |
| **Number of babies** | 1 ± 1 | 1 ± 1 | 2 ± 1 | 2 ± 1 | <0.001 |
| **Gender of the baby** |  |  |  |  | 0.6 |
| Male | 517 (53%) | 283 (52%) | 117 (50%) | 108 (56%) |  |
| Female | 461 (47%) | 264 (48%) | 119 (50%) | 86 (44%) |  |
| **Health condition of the baby** |  |  |  |  | 0.004 |
| Healthy | 930 (95%) | 506 (93%) | 215 (91%) | 180 (93%) |  |
| General | 48 (4.9%) | 37 (6.8%) | 19 (8.1%) | 11 (5.7%) |  |
| Poor | 0 (0%) | 4 (0.7%) | 2 (0.8%) | 3 (1.5%) |  |
| **Household registration of the baby** |  |  |  |  | 0.2 |
| Urban | 631 (65%) | 331 (61%) | 138 (58%) | 126 (65%) |  |
| Rural | 347 (35%) | 216 (39%) | 98 (42%) | 68 (35%) |  |
| **Job of father** |  |  |  |  | <0.001 |
| no job | 123 (13%) | 72 (13%) | 33 (14%) | 31 (16%) |  |
| others | 313 (32%) | 188 (34%) | 81 (34%) | 64 (33%) |  |
| farmer | 18 (1.8%) | 17 (3.1%) | 18 (7.6%) | 15 (7.7%) |  |
| medical related job or teacher or civil servant | 524 (54%) | 270 (49%) | 104 (44%) | 84 (43%) |  |
| **Education of father** | 3 ± 1 | 2 ± 1 | 2 ± 1 | 2 ± 1 | 0.013 |
| **Job of mother** |  |  |  |  | <0.001 |
| no job | 212 (22%) | 126 (23%) | 55 (23%) | 59 (30%) |  |
| others | 210 (21%) | 121 (22%) | 50 (21%) | 42 (22%) |  |
| farmer | 18 (1.8%) | 10 (1.8%) | 15 (6.4%) | 14 (7.2%) |  |
| medical related job or teacher or civil servant | 538 (55%) | 290 (53%) | 116 (49%) | 79 (41%) |  |
| **Education of mother** | 3 ± 1 | 2 ± 1 | 2 ± 1 | 2 ± 1 | <0.001 |
| **Family income** | 3 ± 1 | 3 ± 1 | 3 ± 1 | 3 ± 1 | <0.001 |
| **Religious belief of family members** |  |  |  |  | 0.5 |
| None religous | 759 (78%) | 428 (78%) | 194 (82%) | 153 (79%) |  |
| Religous | 219 (22%) | 119 (22%) | 42 (18%) | 41 (21%) |  |
| **Age in months of the baby** | 39 ± 32 | 40 ± 30 | 47 ± 31 | 44 ± 33 | <0.001 |
| **Age of father** | 34 ± 5 | 34 ± 5 | 35 ± 6 | 36 ± 5 | <0.001 |
| **Age of mother** | 33 ± 5 | 33 ± 5 | 34 ± 5 | 34 ± 5 | 0.001 |
| ^1^Mean ± SD; n (%) | | | | | |
| ^2^Kruskal-Wallis rank sum test; Pearson's Chi-squared test; Fisher's exact test | | | | | |

**Supplemental Table S10.** Distributions of random intercepts and slopes (Best Linear Unbiased Predictors, BLUPs) by K-means clustering and vaccine

| **Vaccine** | **Cluster** | **N^1^** | **Individual params** | **min** | **median** | **max** | **sd** |
| --- | --- | --- | --- | --- | --- | --- | --- |
| DTaP-IPV-Hib | clust 1 | 542 | inter | 3.14 | 4.50 | 5.00 | 0.58 |
| DTaP-IPV-Hib | clust 1 |  | slope | -1.38 | -1.07 | -0.93 | 0.14 |
| DTaP-IPV-Hib | clust 2 | 380 | inter | 0.37 | 1.06 | 2.45 | 0.77 |
| DTaP-IPV-Hib | clust 2 |  | slope | -1.25 | -0.46 | -0.16 | 0.21 |
| DTaP-IPV-Hib | clust 3 | 470 | inter | -2.93 | -1.76 | 0.16 | 1.00 |
| DTaP-IPV-Hib | clust 3 |  | slope | -0.53 | -0.02 | 0.45 | 0.29 |
| DTaP-IPV-Hib | clust 4 | 557 | inter | -6.85 | -6.07 | -3.93 | 1.22 |
| DTaP-IPV-Hib | clust 4 |  | slope | -0.02 | 0.87 | 1.12 | 0.23 |
| EV71 | clust 1 | 853 | inter | 1.64 | 3.74 | 3.74 | 0.57 |
| EV71 | clust 1 |  | slope | -1.07 | -0.81 | -0.81 | 0.14 |
| EV71 | clust 2 | 555 | inter | -1.97 | -1.20 | 0.40 | 0.99 |
| EV71 | clust 2 |  | slope | -0.58 | -0.10 | 0.27 | 0.23 |
| EV71 | clust 3 | 268 | inter | -5.08 | -4.33 | -2.61 | 0.95 |
| EV71 | clust 3 |  | slope | 0.07 | 0.63 | 0.99 | 0.26 |
| EV71 | clust 4 | 278 | inter | -8.30 | -7.63 | -5.82 | 1.03 |
| EV71 | clust 4 |  | slope | 0.54 | 1.29 | 1.48 | 0.22 |
| Influenza | clust 1 | 978 | inter | 0.96 | 3.30 | 3.30 | 0.58 |
| Influenza | clust 1 |  | slope | -0.88 | -0.70 | -0.70 | 0.13 |
| Influenza | clust 2 | 547 | inter | -2.65 | -1.62 | -0.36 | 1.04 |
| Influenza | clust 2 |  | slope | -0.93 | 0.01 | 0.38 | 0.26 |
| Influenza | clust 3 | 236 | inter | -5.99 | -5.20 | -3.85 | 0.89 |
| Influenza | clust 3 |  | slope | -0.37 | 0.83 | 1.24 | 0.31 |
| Influenza | clust 4 | 194 | inter | -8.61 | -8.26 | -6.62 | 0.88 |
| Influenza | clust 4 |  | slope | 0.36 | 1.39 | 1.54 | 0.31 |
| Varicella | clust 1 | 1035 | inter | 0.94 | 3.06 | 3.06 | 0.58 |
| Varicella | clust 1 |  | slope | -0.68 | -0.68 | -0.68 | 0.14 |
| Varicella | clust 2 | 488 | inter | -2.59 | -1.93 | -0.19 | 0.99 |
| Varicella | clust 2 |  | slope | -0.72 | 0.04 | 0.43 | 0.25 |
| Varicella | clust 3 | 228 | inter | -5.70 | -4.87 | -3.36 | 0.93 |
| Varicella | clust 3 |  | slope | -0.13 | 0.77 | 1.15 | 0.32 |
| Varicella | clust 4 | 204 | inter | -9.01 | -8.24 | -6.54 | 1.00 |
| Varicella | clust 4 |  | slope | 0.59 | 1.48 | 1.67 | 0.24 |

^1^ Number of households in the corresponding cluster.
